# Integrating Soil Silicon Amendment into Management Programs for Insect Pests of Drill-Seeded Rice

**DOI:** 10.3390/plants6030033

**Published:** 2017-08-13

**Authors:** James M. Villegas, Michael O. Way, Rebecca A. Pearson, Michael J. Stout

**Affiliations:** 1 Department of Entomology, Louisiana State University Agricultural Center, 404 Life Sciences Building, Baton Rouge, LA 70803, USA; jvillegas@agcenter.lsu.edu; 2 Texas A&M AgriLife Research and Extension Center, 1509 Aggie Drive, Beaumont, TX 77713, USA; moway@aesrg.tamu.edu (M.O.W.); rawolff@ag.tamu.edu (R.A.P.)

**Keywords:** silicon amendment, rice water weevil, stem borers, seed treatment, host-plant resistance

## Abstract

Silicon soil amendment has been shown to enhance plant defenses against insect pests. Rice is a silicon-accumulating graminaceous plant. In the southern United States, the rice water weevil and stem borers are important pests of rice. Current management tactics for these pests rely heavily on the use of insecticides. This study evaluated the effects of silicon amendment when combined with current management tactics for these rice insect pests in the field. Field experiments were conducted from 2013 to 2015. Rice was drill-planted in plots subjected to factorial combinations of variety (conventional and hybrid), chlorantraniliprole seed treatment (treated and untreated), and silicon amendment (treated and untreated). Silicon amendment reduced densities of weevil larvae on a single sampling date in 2014, but did not affect densities of whiteheads caused by stem borers. In contrast, insecticidal seed treatment strongly reduced densities of both weevil larvae and whiteheads. Higher densities of weevil larvae were also observed in the hybrid variety in 2014, while higher incidences of whiteheads were observed in the conventional variety in 2014 and 2015. Silicon amendment improved rice yields, as did chlorantraniliprole seed treatment and use of the hybrid variety.

## 1. Introduction

Silicon (Si) is the second most abundant element, after oxygen, in the earth’s crust, and is almost exclusively found in the form of silicon dioxide (SiO_2_), in association with a wide array of Si-bearing minerals in crystalline, poorly crystalline, and amorphous phases [1,2]. In the soil solution, silicon is mainly present in the form of silicic acid, H_4_SiO_4_, the only form of water-soluble silicon absorbed by plants. Silicon is taken up by plants by the lateral roots via active, passive, and rejective mechanisms [3], and is transported to the shoot as monosilicic acid, where it is deposited as solid, amorphous, hydrated silica (SiO_2_.nH_2_O) known as pytoliths [4,5]. Monocotyledons such as wheat, sugarcane, rice, and barley are classified as high accumulators of silicon containing 10–100 g kg^−^ Si in dry weight [6,7,8]. Also, some dicotyledonous plant species such as those belonging to the Cucurbitaceae and Fabaceae are able to accumulate silica concentrations which are higher or almost as high as those found in grass species [9]. Although silicon is not classified as an essential plant nutrient, it is considered a beneficial nutrient for rice [7]. Recently, the International Plant Nutrition Institute, Georgia, USA listed silicon as a “beneficial substance” [10]. Silicon application can mitigate both biotic and abiotic plant stresses [11]. Biotic stressors may come in the form of plant pathogens and animals (vertebrate and arthropod herbivores) [12]. Silicon can enhance plant defenses via physical and induced biochemical mechanisms. Silicon may protect plants by reducing insect performance or plant injury and delaying herbivore establishment, which increases the chance of herbivore exposure to natural enemies (e.g., parasitoids and predators), adverse climatic conditions (e.g., dispersal to unfavorable microhabitats and desiccation), or control measures that target exposed insects [13]. Apart from enhancing plant resistance, the addition of silicon to the soil has been shown to directly increase the growth and grain yield in rice plants by increasing the numbers of panicles and spikelets per panicle and decreasing blank spikelets [14].

Rice (*Oryza sativa* L.) is one of the most important crops globally. It is a staple for nearly half of the world’s seven billion people [15]. The United States is a major rice producer with an acreage of 3.15 M acres and a production of 14.4 M tons in 2016 [16]. About half of the rice produced in the United States is exported [17]. Rice plants are subjected to various pressures by pests and diseases throughout the planting season. Consistently, the rice water weevil, *Lissorhoptrus oryzophilus* Kuschel (Coleoptera: Curculionidae), is the most destructive and widely distributed early-season insect pest of rice in the United States [18,19]. This insect poses a global threat to rice production, having recently invaded rice-producing regions of Asia and Europe [20]. Yield losses due to rice water weevil can exceed 25% under heavy infestations [21]. Rice water weevil adults overwinter in leaf litter, bunch grasses, and stubble in and around rice fields, and emerge in early spring [22]. Adults feed on the young leaves of rice plants, leaving narrow longitudinal scars parallel to the venation of the leaves [23]. Injury from adult feeding is generally not economically important. Flooding of rice fields triggers female weevils to oviposit, primarily in leaf sheaths beneath the water surface [24]. Rice water weevil larvae may feed on the rice leaves and stems shortly after eclosion but soon move down to the roots to feed until pupation. Root feeding by the larvae can cause significant yield loss due to reduced growth during vegetative stages [25] and reduction in tillers and grains per panicle at maturity [26]. 

Moreover, complexes of stem boring Lepidopterans that attack rice plants from the seedling stage to maturity [27] are important rice insect pests worldwide. The sugarcane borer, *Diatraea saccharalis* F. (Lepidoptera: Crambidae), and, sporadically, the rice stalk borer, *Chilo plejadellus* Zincken (Lepidoptera: Crambidae), are economically important stem boring pests of rice in Texas and Louisiana [28], while an invasive species, the Mexican rice borer, *Eureuma loftini* Dyar (Lepidoptera: Crambidae), is causing increasing yield loss in Texas and Louisiana [29]. Stem borer larvae initially feed on the leaf sheath for a few days before boring into the stem. When feeding occurs during the vegetative phase of the plant, the central leaf whorl does not unfold, but turns brownish and dries off, and the affected tillers dry out without bearing panicles, a condition known as a deadheart [30]. When feeding occurs after panicle initiation, severance of the growing plant parts from the base dries the panicles, which may not emerge; panicles that have emerged do not produce grains but remain straight and are whitish, a condition known as a whitehead [30]. There appears to be a negative correlation between the number of whiteheads and the main crop yield [31]; thus, stem borer activity in the rice field is often measured through whitehead incidence. 

Ideally, the management of rice water weevil and stem boring pests should incorporate combinations of control tactics such as host plant resistance, insecticide application, and cultural practices. However, current management programs for these rice insect pests rely heavily on chemical insecticides [18,32]. Seed treatments such as the anthranilic diamide chlorantraniliprole (Dermacor X-100, DuPont Crop Protection, Wilmington, DE) have been the most effective management tactic in reducing densities of rice water weevil [33,34] and can also reduce the performance of stem borers and whitehead incidence in the field [35,36]. Cultural control methods that involve field draining, weed control, and delayed flooding have also been utilized to manage the rice water weevil [37,38]. Although some level of resistance and tolerance traits have been observed in some rice cultivars against the rice water weevil [39], attempts to incorporate these traits have not been successful to date [40]. Another control tactic for managing stem borer infestations and yield losses in Texas has been the use of foliar applications of pyrethroids at the reproductive and late boot or early heading phases of rice development [32]. Although Texas rice cultivars showed varying levels of injury and yield loss from stem borers, they are not currently bred for resistance to stem borers [31,41]. In Asia, rice cultivars are screened for resistance to stem borers, and the use of resistant cultivars combined with cultural control strategies are now the main tactics under development for stem borer management [42].

In this study, we evaluated the effects of soil silicon amendment when combined with insecticide treatment and rice varieties (conventional and hybrid) for the management of rice insect pests. Although a number of studies have shown the adverse effects of silicon soil amendment on insect pests in rice and other cereals, there is a lack of field studies showing the effect of silicon on insect pests, and a lack of studies investigating the effects of silicon on root feeders. This study provides the first report on the effect of silicon on a root feeding pest of rice, the rice water weevil. This is also one of the few field studies that investigated the effects of silicon soil amendment on natural field infestations of multiple pests in rice.

## 2. Results

### 2.1. Effects on Plant Stand

**Figure 1:**
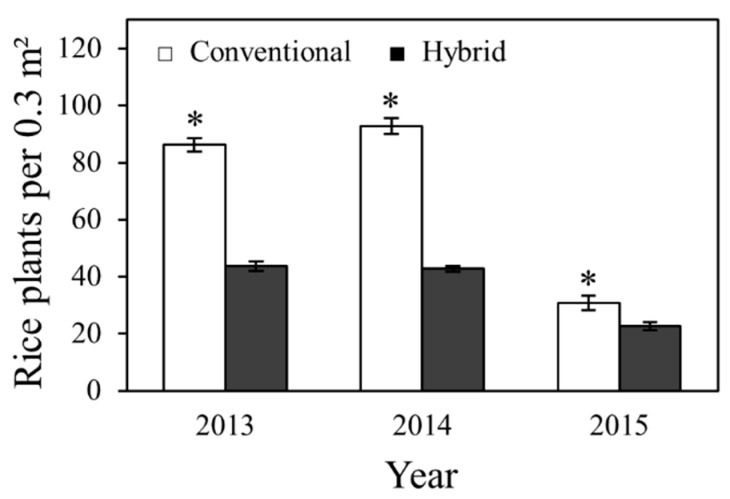
Mean rice stand per 0.3 m^2^ (±SEM) of conventional and hybrid varieties in 2013, 2014, and 2015. An asterisk denotes a significant main effect of variety (*P* < 0.05; Tukey’s HSD).

In this study, significantly higher numbers of rice plants per 0.3 m^2^ were observed in plots of the conventional varieties compared to hybrid varieties in 2013 (*F* = 224.40; df = 1, 23; *P* < 0.0001), 2014 (*F* = 344.83; df = 1, 23; *P* < 0.0001), and 2015 (*F* = 7.09; df = 1, 24; *P* = 0.0136) (Figure 1). The difference in plant stand between varieties was most likely caused by the use of lower seeding rates for hybrid varieties. Conventional varieties were drill-seeded at seeding rates of 90–100 kg/ha, while hybrid varieties were drill-seeded at seeding rates of 23–28 kg/ha, as per the recommended seeding rates provided in the Texas Rice Production Guidelines [43]. Dermacor seed treatment did not affect stand counts in 2013 (*F* = 0.53; df = 1, 23; *P* = 0.4752), 2014 (*F* = 1.28; df = 1, 23; *P* = 0.2690), or 2015 (*F* = 0.01; df = 1, 24; *P* = 0.9373). Similarly, amendment of soils with calcium silicate (slag) did not affect stand counts in 2013 (*F* = 3.66; df = 1, 23; *P* = 0.0682), 2014 (*F* = 1.15; df = 1, 23; *P* = 0.2950), or 2015 (*F* = 1.10; df = 1, 24; *P* = 0.3049). Furthermore, there were no significant interactions between variety, Dermacor and silicon treatments (data not shown).

### 2.2. Effects on Rice Water Weevil Larval Density

**Figure 2:**
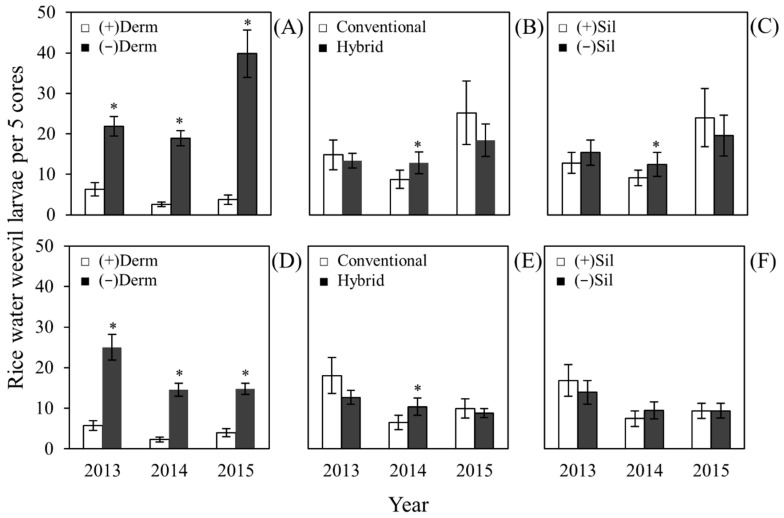
Main effects of Dermacor seed treatment, variety, and silicon amendment on rice water weevil larval densities. Core 1 (**A**, **B** and **C**) and Core 2 (**D**, **E** and **F**) sampling were performed three and four weeks after permanent flooding. (**A** and **D**), main effect of Dermacor treatment; (**B** and **E**), main effect of variety; (**C** and **F**), main effect of silicon amendment in 2013, 2014, and 2015. For each year, bars accompanied by asterisks designate significant differences (*P* < 0.05; Tukey’s HSD). The bars represent standard error of means (SEM).

**Figure A1:**
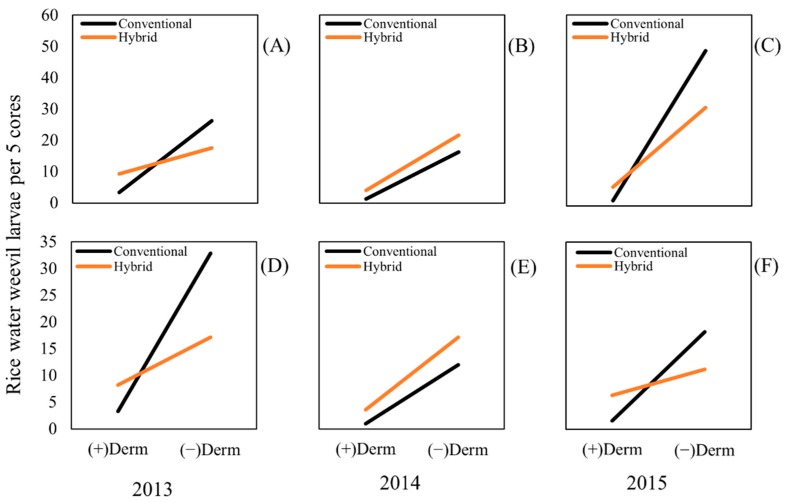
Interactive effects of Dermacor treatment and variety on *L. oryzophilus* larval densities in 2013, 2014, and 2015. Core 1 (**A**, **B** and **C**) and Core 2 (**D**, **E** and **F**) sampling were performed three and four weeks after permanent flooding. Parallel lines indicate absence of interaction (*P* > 0.05), while lines with different slopes (non-parallel lines) indicate presence of interaction (*P* < 0.05). Figure A,C,D,F show significant interactions.

The larval stage is the most destructive developmental stage of the rice water weevil. Root feeding by weevil larvae can cause significant yield losses [25,26]. Core samplings were performed three and four weeks after permanent flooding to determine larval densities in the field. Data were analyzed separately by core sampling and year. In both the first and second core samplings, larval densities were significantly reduced (*P* < 0.05) in Dermacor-treated rice plots in 2013, 2014, and 2015, compared to untreated rice plots (Figure 2A,D and Table 1). The effect of variety on weevil densities was not significant in 2013 and 2015; however, significantly higher (*P* < 0.05) weevil densities were observed on the hybrid variety in both core samplings in 2014 (Figure 2B,E and Table 1). Weevil densities were not affected by silicon amendment in 2013 and 2015 (Table 1). In 2014, significantly lower larval densities (*P* < 0.05) were observed in silicon-treated plots in the first core sampling but not the second (Figure 2C,F). There was a significant interaction (*P* < 0.05) between Dermacor treatment and variety in the first and second core samplings in 2013 and 2015 (Table 1). The interaction between Dermacor treatment and variety in 2013 and 2015 suggests that when rice plots are left untreated with Dermacor, rice water weevil larval densities tend to be higher in plots seeded with the conventional variety than hybrid plots (Figure A1, in Appendix A). When rice seeds were treated with Dermacor, reductions in rice water weevil larval densities are similar between varieties. 

### 2.3. Effects on Whitehead Incidence

**Figure 3:**
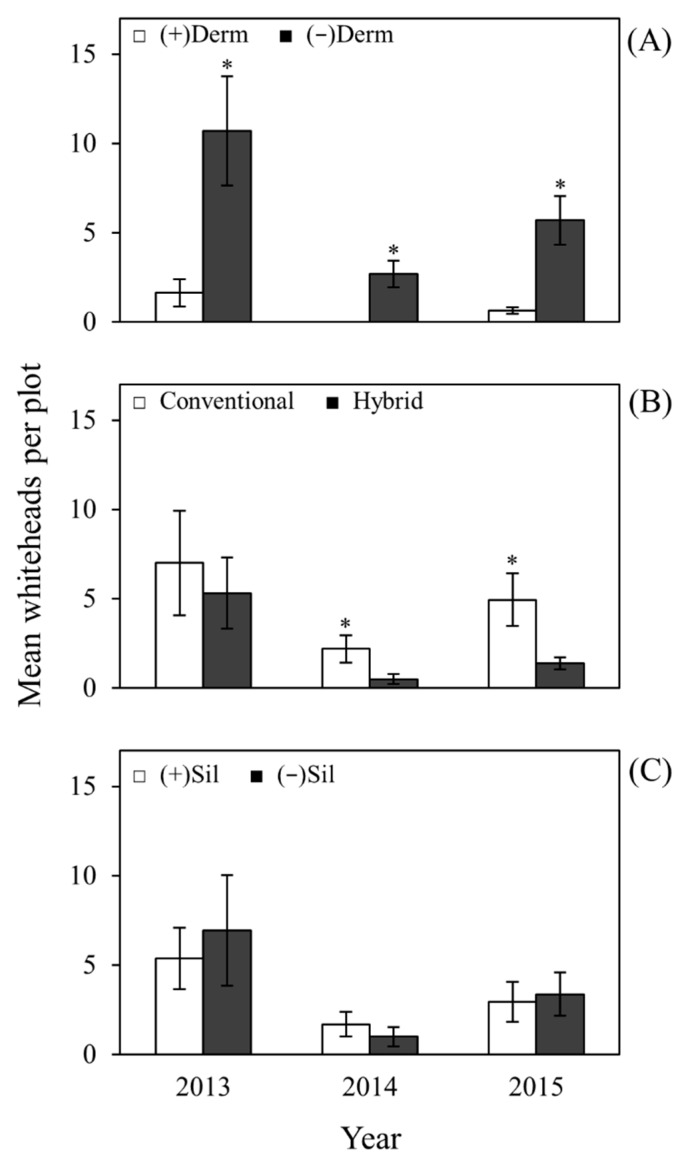
Main effects of Dermacor seed treatment, variety, and silicon amendment on whiteheads. (**A**) main effect of Dermacor treatment; (**B**) main effect of variety; (**C**) main effect of silicon amendment in 2013, 2014, and 2015. For each year, bars accompanied by asterisks designate significant difference (*P* < 0.02; Tukey’s HSD). The bars represent standard error of means (SEM).

**Figure A2:**
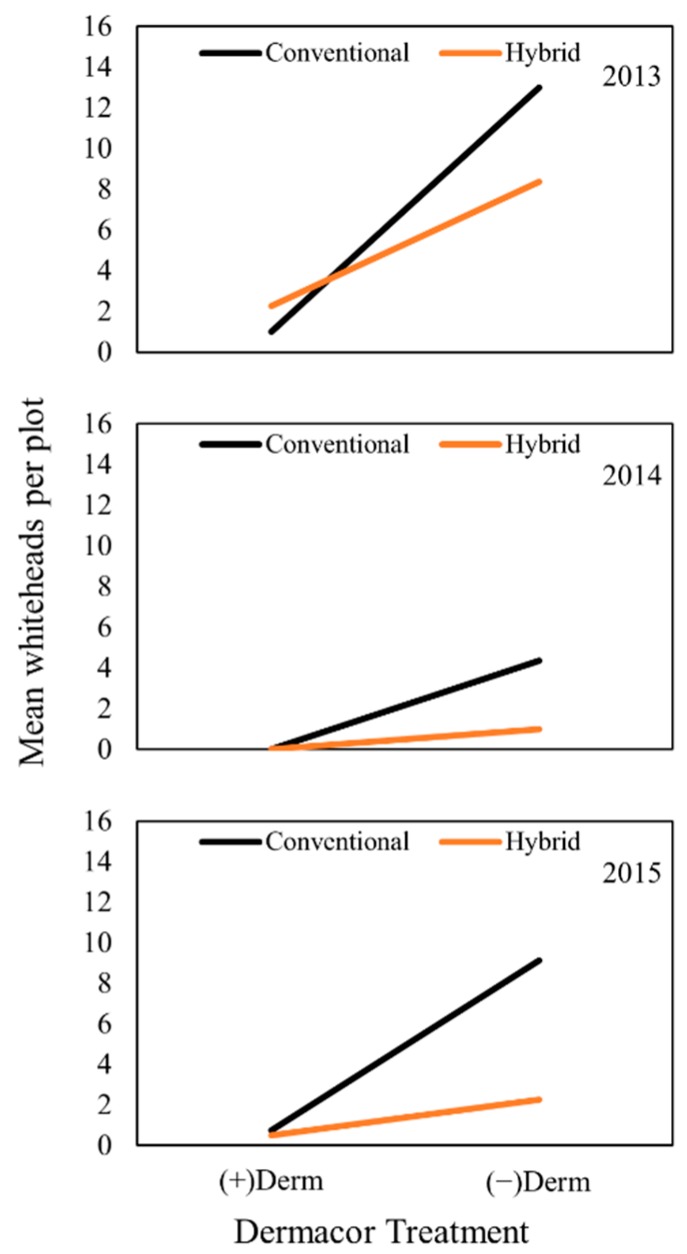
Interactive effects of Dermacor treatment and variety on whitehead densities in 2013, 2014, and 2015. Parallel lines indicate absence of interaction (*P* > 0.05), while lines with different slopes (non-parallel lines) indicate presence of interaction (*P* < 0.05). Figures 2014 and 2015 show significant interactions.

Densities of whiteheads (whiteheads per plot) were used as an indicator of stem borer activity in the field. Whiteheads were collected and subsequently dissected to identify the stem borer species. Mexican rice borer was found to be the major cause of whiteheads in the field. In this study, Dermacor seed treatment significantly reduced whitehead incidence in treated rice plots compared to untreated rice plots in 2013 (*F* = 18.14; df = 1, 24; *P* = 0.0003), 2014 (*F* = 24.64; df = 1, 23; *P* < 0.0001), and 2015 (*F* = 41.79; df = 1, 23; *P* < 0.0001) (Figure 3A). Significantly lower whitehead incidence was also observed in the hybrid variety compared to the conventional variety in 2014 (*F* = 9.71; df = 1, 23; *P* = 0.0049) and 2015 (*F* = 9.32; df = 1, 23; *P* = 0.0056), but not in 2013 (*F* = 0.05; df = 1, 24; *P* = 0.8256) (Figure 3B). Soil silicon amendment did not affect whitehead incidence in 2013 (*F* = 1.53; df = 1, 23; *P* = 0.2283), 2014 (*F* = 1.61; df = 1, 23; *P* = 0.2169), or 2015 (*F* = 0.03; df = 1, 23; *P* = 0.8591) (Figure 3C). There was a significant interaction between variety and Dermacor in 2014 (*F* = 9.71; df = 1, 23; *P* = 0.0049) and 2015 (*F* = 6.96; df = 1, 23; *P* = 0.0147) but not in 2013 (*F* = 2.90; df = 1, 24; *P* = 0.1012). The interaction observed between Dermacor treatment and variety in 2014 and 2015 is probably attributable to the higher whitehead incidence in the conventional variety than in the hybrid variety when left untreated with Dermacor (Figure A2, in Appendix A). When rice plots are treated with Dermacor, reductions in whiteheads are similar between varieties.

### 2.4. Effects on Yield

**Figure 4:**
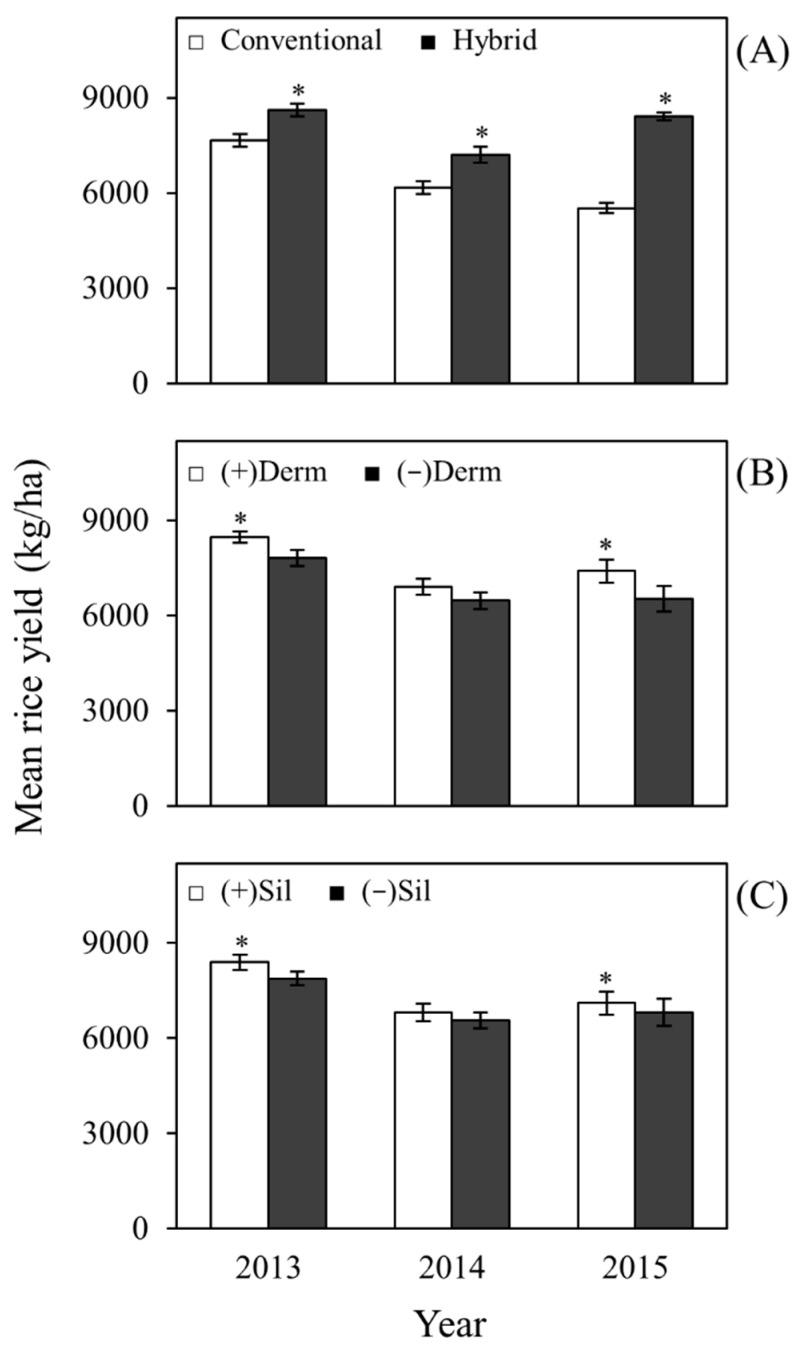
Main effects of variety, Dermacor seed treatment, and silicon amendment on rice yields (kg/ha ± SEM). (**A**) main effect of variety; (**B**) main effect of Dermacor treatment; (**C**) main effect of silicon amendment in 2013, 2014, and 2015. For each year, bars accompanied by asterisks designate significant difference (*P* < 0.05; Tukey’s HSD). The bars represent standard error of means (SEM).

Rice yields from hybrid varieties were consistently higher compared to conventional varieties. Yields from hybrid plots were higher by 969 kg/ha in 2013 (*F* = 17.77; df = 1, 23; *P* = 0.0003), 1042 kg/ha in 2014 (*F* = 12.46; df = 1, 23; *P* = 0.0018), and 2899 kg/ha in 2015 (*F* = 629.19; df = 1, 23; *P* < 0.0001) (Figure 4A). Dermacor treatment significantly increased rice yields by 657 kg/ha in 2013 (*F* = 8.16; df = 1, 23; *P* = 0.0089) and 879 kg/ha in 2015 (*F* = 57.79; df = 1, 23; *P* < 0.0001) but did not significantly increase rice yields in 2014 (*F* = 2.18; df = 1, 23; *P* = 0.1536 (Figure 4B). Similarly, rice yields in silicon-treated plots were higher by 517 kg/ha in 2013 (*F* = 5.05; df = 1, 23; *P* = 0.0345) and 298 kg/ha in 2015 (*F* = 6.65; df = 1, 23; *P* = 0.0168) but not significantly higher in 2014 (*F* = 0.70; df = 1, 23; *P* = 0.4105) (Figure 4C). Furthermore, there was a significant interaction between variety and silicon (*F* = 5.69; df = 1, 23; *P* = 0.0257) in 2015 but not in 2013 (*F* = 0.70; df = 1, 23; *P* = 0.4105) or 2014 (*F* = 0.25; df = 1, 23; *P* = 0.6248). The interaction between silicon and variety in 2015 suggests that the effect of silicon amendment on yield was greater in plots with the conventional variety than in hybrid plots (data not shown). 

## 3. Discussion

To ensure sustainable food production, it is essential to combine management tactics and not rely solely on the application of insecticides. Ideally, the management of rice water weevil and stem boring pests in rice should incorporate combinations of control tactics such as host plant resistance, insecticide application, and cultural practices. This study was conducted to investigate the effects of integrating soil silicon amendment with current management and production practices (chemical control and variety) for managing these pests. This study is among the first to investigate the effects of silicon amendment on root feeding herbivores under field conditions. To our knowledge, the only other study to report reductions in the performance of a root-feeding insect in response to silicon was conducted on sugarcane using soil-dwelling ‘canegrubs’ [44]. 

Amendment of soils with silicon has been proposed to augment plant resistance via two mechanisms [13]. Uptake of silicon by plants can lead to deposition of silica and thereby contribute to the thickening of the epidermal layer and increases in the rigidity and abrasiveness of plant tissues, thus forming a mechanical barrier and reducing the palatability and digestibility of plants to insect herbivores [45,46,47]. Silicon may also stimulate biochemical pathways related to resistance against biotic stresses such as borers, hoppers, and mites [48,49,50]. In this study, densities of root-feeding rice water weevil larvae were affected by silicon amendment in only one core sampling in the 2014 experiment (Table 1). The phenology of the rice water weevil attack could be one of the reasons for the weak effect of soil silicon amendment. Rice water weevils attack rice plants early in the planting season. Silicon content in rice plants varies with plant age, with older plants and leaves typically having higher silicon content than younger plants and leaves due to continual uptake by roots and the immobility of silicon once deposited in leaves and stems [51]. Thus, silicon may not have accumulated to a sufficient level in young rice plants to consistently influence the attack by rice water weevils. Rice variety might also have been a factor, as different varieties commonly grown in the southern United States accumulate silicon at significantly different rates [52]. Furthermore, the amount of monosilicic acid released from the calcium silicate slag is highly influenced by the adsorption capacity of soils, which is determined by the soil pH, organic matter, and clay content [53]. 

Silicon amendment also did not affect whitehead incidence in the field in 2013, 2014, or 2015 (Figure 3). This was unexpected because other studies have shown the effects of silicon on stem borers in rice. In fact, the first report of silicon-induced insect pest resistance was associated with resistance against the rice stem borer, *Chilo simplex* [54]. Since that time, the use of silicon has been documented to enhance plant resistance to several rice insect pests such as yellow stem borer [55], green rice caterpillar [56], Asiatic rice borer [49], sugarcane borer [57], and other insect pests. Silicon amendment also decreased the penetration, weight gain, and stem damage, and prolonged the penetration time and larval development of the Asiatic rice borer, *Chilo suppresalis* Walker, a destructive stem boring rice pest in Asia [49]. The lack of effect of silicon on whitehead incidence in the field in this study could be attributed to several factors. To begin with, the sole use of whiteheads as an indicator of stem borer activity in the field may have provided an incomplete picture of the effects of silicon amendment on resistance to borers. Other types of stem borer injury such as deadhearts (destruction of the apical part of stalk), partial whiteheads (only a portion of the grains on the panicle are consumed by larvae), and unemerged whiteheads [42] may also have been present and should also be evaluated in the field in future experiments. In addition, the varieties used in the experiment may not have taken up silicon sufficiently to influence infestation by stem borers, or the uneven distribution and relatively low populations of stem borers in the field may have prevented detection of a silicon effect. Finally, the borer species present in the field in these experiments may be less sensitive to silicon-induced changes than previously investigated species. In prior greenhouse experiments, silicon soil amendment led to lower relative growth rates and boring success of sugarcane borer [57], but the majority of borers present in the field in these experiments were Mexican rice borers.

Despite the lack of effects of silicon on insects, yields were affected by silicon treatment. Silicon amendment significantly improved rice yields in 2013 and 2015 (Figure 4) by 6.6% and 4.4%, respectively, compared to untreated plots. Beneficial effects of silicon on crop yield have been previously documented. Field trials conducted in Japan has resulted in an increase in the number of panicles and an up to 17% increase in rice yield due to silicon fertilization [7]. Application of silicon-containing materials also increased grain yield in wheat by 4.1–9.3% [58]. The increases in yield due to silicon (4.4–6.6%) were smaller than the increases due to use of Dermacor (8.4–13.5%) and the increases observed in hybrid plots over conventional plots (12.7–52.5%).

Chlorantraniliprole seed treatment (Dermacor X-100) consistently reduced densities of *L. oryzophilus* larvae (Figure 1, Table 1) and the incidence of whiteheads (Figure 3) in treated field plots. Chlorantraniliprole belongs to a newer class of insecticides called anthranilic diamides that function by disrupting ryanodine receptors, leading to an uncontrolled calcium ion release from the sarcoplasmic reticulum of muscle cells that cause paralysis and death in insects [59]. It is a systemic insecticide that persists in plants for long periods of time [60]. High residues of chlorantraniliprole have been found in the rice roots, causing a strong impact on root-feeding larvae [61]. Field studies have shown reductions of 80–94% on weevil larval densities in rice treated with chlorantraniliprole in both Louisiana and Texas [62], a level of reduction consistent with what was observed in this study. Previous studies have demonstrated that chlorantraniliprole can suppress rice water weevil oviposition and can affect the survival of eggs or first instars [61,63] but does not affect the survival of adults feeding on the leaves of seed-treated plants [64]. Chlorantraniliprole has also demonstrated an exceptional activity across a broad range of pests in the order Lepidoptera [60]; however, there is a limited published literature on the effects of chlorantraniliprole seed treatment on stem boring pests in rice fields. Field trials conducted in Texas reported reductions on whitehead densities in chlorantraniliprole-treated fields [35] similar to the reductions observed in this study. A greenhouse experiment, in which sugarcane borer larvae were left to feed on rice plants treated with Dermacor, resulted in a 70–80% mortality rate [36]. Due to the effective control of rice water weevil and stem boring pests, chlorantraniliprole seed treatment significantly increased rice yields in 2013 and 2015 by 657 kg/ha and 879 kg/ha, respectively (Figure 4).

Rice water weevil densities were lower in plots planted with the conventional variety ‘Antonio’ than in plots planted with the hybrid variety ‘XL753’ in 2014, and lower incidences of whiteheads were observed in plots planted with ‘XL753’ than in plots planted with ‘Antonio’ in both 2014 and 2015 (Figure 2 and Figure 3). One possible explanation for these differences is, of course, that ‘Antonio’ and ‘XL753’ differ in their inherent resistance to rice water weevils and Mexican rice borers. Prior studies have shown that some of the cultivars commonly grown in the southern United States do differ in their susceptibility to infestation by rice water weevil larvae and their tolerance of rice water weevil feeding, although none possess high levels of resistance [39]. ‘Antonio’ is a long-grain variety released in 2012 that was derived from a cross of the conventional inbred varieties ‘Cocodrie’ and ‘Cypress‘ [43]. Although ‘Antonio’ has been available for commercial planting since 2014, it has not previously been evaluated for resistance to the rice water weevil. The conventional varieties ‘Cocodrie’ and ‘Cypress’, on the other hand, were found to support high densities of rice water weevil larvae, and ‘Cypress’ exhibited poor tolerance to weevil injury compared to other varieties [39]. In addition, based on field trials conducted in 2015 and 2016, levels of infestations of rice water weevil larvae on the long-grain hybrid variety ‘XL753’ have been found to be similar to those found on other commonly grown conventional varieties in Louisiana ([65], unpublished). Oviposition preference and larval performance of sugarcane borers have also been found to differ among rice cultivars in greenhouse studies [66,67], although ‘Antonio’ and ‘XL753’ have not been evaluated. Furthermore, a four-year field study that was conducted in Texas to evaluate varietal resistance to stem borers showed that hybrid cultivars were among the cultivars with the lowest number of whiteheads [31], consistent with what was observed in this study. Thus, it is likely that the differences in weevil densities and whitehead incidences observed in plots planted with ‘Antonio’ and ‘XL753’ in this study are attributable in part to differences in inherent varietal resistance between these two varieties.

Differences in inherent varietal resistance to rice water weevils and stem borers are not, however, the only possible explanation for differences in weevil densities and whitehead incidence between ‘Antonio’ and ‘XL753’ plots in 2014 and 2015. In order to reflect recommended agronomic practices for seeding rates and nitrogen fertilization (based on the Texas Rice Production Guidelines [43]), plots planted with hybrid varieties were seeded at a much lower rate and were fertilized at a higher rate than plots planted with conventional varieties. The higher rice water weevil densities in the hybrid variety in 2014 might therefore be due to the lower seeding rate and higher nitrogen fertilization used in these plots. A previous study showed that the low seeding rate increases the vulnerability of rice to infestation by rice water weevils [38]. Furthermore, rice water weevil infestations tend to increase with nitrogen fertilization [68]. These differences in cultural practices do not, however, explain the lower whitehead incidences in hybrid plots in 2014 and 2015, but the effects of seeding rate and N fertilization on Mexican rice borer and sugarcane borer densities have not been previously investigated in U.S. rice. Injuries from Mexican rice borer were higher at higher nitrogen rates on bioenergy sorghum [69]. Similarly, injury from sugarcane borer increases as nitrogen rates increase in sugarcane [70]. 

There is an apparent lack of studies on the effects of silicon on below ground herbivores, and most prior studies have been conducted in greenhouse and laboratory environments. There is a need to elucidate the effects of silicon on insect pests in field conditions. This study is among the first to investigate the role of silicon in augmenting the resistance of rice against multiple insect pests in the field, and also among the first to document the effects of silicon on a root-feeding pest in the field. The effect of silicon soil amendment was found to be weaker than both the effect of insecticidal seed treatment and variety. It is important to understand the role of silicon amendment as a potential component of control strategies against rice pests in the southern United States. Despite the weak effect of silicon on insect pests in this study, silicon could still play an important role in rice production considering the positive effects on yield and the documented effects on disease suppression. 

## 4. Materials and Methods 

Field experiments were conducted from 2013 to 2015 at the Texas A&M AgriLife Research and Extension Center in Beaumont, TX to investigate the interactive effects of rice variety, chlorantraniliprole seed treatment, and soil silicon amendment on rice plant stand, *L. oryzophilus* larval density, whitehead incidence, and rice yield. Experiments employed a randomized complete block design with four replications. Each block consisted of eight plots subjected to factorial combinations of the following treatments: two varieties (conventional and hybrid), two levels of chlorantraniliprole seed treatment (treated and untreated), and two levels of silicon slag applications (treated and untreated). Rice was drill-planted in plots measuring 5.5 m long and plots with seven rows spaced 18 cm apart in League soil (pH 5.5, sand 3.2%, silt 32.4%, clay 64.4% and organic matter 3.8–4.8%). In 2013, rice was planted at seeding rates of 100 kg/ha for the conventional long-grain variety ‘Cocodrie’ and 28 kg/ha for the hybrid variety ‘XL723’. In 2014 and 2015, rice was planted at seeding rates of 90 kg/ha for the conventional long-grain ‘Antonio’ and 23 kg/ha for the hybrid ‘XL753’. For plots assigned to silicon treatments, silicon slag (Ca_2_SiO_4_) was evenly spread on the soil surface of plots at a rate of 4000 kg/ha immediately after planting. Slag was then distributed into the top 2 cm of soil using a rake. For plots assigned to chlorantraniliprole treatments, seeds were treated with the insecticide Dermacor X-100 (chlorantraniliprole, 50%, DuPont, Wilmington, DE) one to four days before seeding at rates of 0.06 kg a.i./ha for conventional varieties and 0.08 kg a.i./ha for hybrid varieties. Seeds were treated with insecticide by hand in small batches following the methods of Lanka et al. [61]. Table 2 lists the dates of planting, seed treatment, and slag application for the three years of the study.

After planting, fields were surface irrigated as needed until permanent flooding to facilitate plant emergence and stand establishment. Permanent flood was applied four to five weeks after planting (Table 2). Nitrogen was applied in the form of urea at 193 kg N/ha for conventional varieties and 205 kg N/ha for hybrid varieties, except in 2013 when the rate was 243 kg N/ha, as per the agronomic recommendations provided in Texas Rice Production Guidelines [43]. For conventional varieties, nitrogen fertilization was divided into three applications: 20% at planting, 50% immediately before permanent flooding, and 30% at panicle differentiation. For hybrid varieties, fertilization was divided into two applications: 66% before permanent flooding and 34% at late boot/early heading, except in 2013, when nitrogen was also applied at planting. Post-emergence herbicides applied for early-season weed management included propanil and thiobencard (Ricebeaux [Riceco, Memphis, TN]) at 1.88 kg a.i./ha, clomazone (Command 3 ME [FMC Corporation, Philadelphia, PA]) at 0.57 kg a.i./ha, and halosulfuron-methyl (Permit [Gowan Company, Yuma, AZ]) at 0.05 kg a.i./ha. Herbicides were applied with a two-person, hand-held spray boom (13-80015 nozzles, 50-mesh screens, 16 GPA final spray volume). 

Plant stands (number of rice plants per 0.3 m^2^) were evaluated approximately two weeks after plant emergence (Table 2). Stand establishment is an important factor in successful rice crop management. The goal is to have a uniform stand of healthy rice seedlings. There are various factors that can affect stand establishment such as cultivar, seeding rate, seed treatment, soil properties, seeding method, environment, and geographic location [71]. Plant stands were determined by counting the number of plants in three 0.9 meter rows in every plot. Rows were randomly selected each year. The total number of plants were summed in each plot for analysis. 

*L. oryzophilus* larval densities were determined using a metal soil/root core sampler with a 10 cm diameter and a 10 cm depth. For each sampling date, five cores were taken from every plot. Each core sample contained a minimum of one rice plant with intact roots. Individual core samples were processed by washing the soil from roots in 40-mesh screen sieve buckets. Larvae were counted as they floated from the sieve buckets when dipped on basins with a salt solution [39]. The larval density in each plot was obtained by summing the number of larvae from five core samples. Core samples were taken twice, approximately three and four weeks after flooding (Table 2). 

An incidence of whiteheads resulting from stem borer infestations of rice in the reproductive stages of development was assessed by counting the total number of whiteheads in four randomly selected rows in each plot. The number of whiteheads in four rows was summed to obtain the total number of whiteheads in each plot. Approximately 20 whiteheads were brought back to the laboratory and dissected to identify the stem borer species. Mexican rice borer was found to be the major cause of whiteheads in the field. 

At grain maturity, each plot was harvested entirely with a small plot rice combine. Grain yields from each plot were adjusted to 12% moisture. 

### Data Analysis

Stand counts, *L. oryzophilus* larval densities, incidence of whiteheads, and yields in 2013, 2014, and 2015 were analyzed separately as a factorial RBD experiment with block as random effect and variety, insecticide treatment, and silicon amendment, and their interactions as fixed effects using the mixed model analysis of variance in PROC MIXED of SAS [72]. Larval densities from the two core sampling dates were also analyzed separately. Means were separated using Tukey’s HSD test [73]. Residuals were analyzed for normality using PROC UNIVARIATE [72]. Transformations to normalize the data distribution to satisfy statistical assumptions were performed as necessary, but untransformed data are presented. Transformations were performed on the following data sets using either square or cube root: core 1, core 2, and whitehead incidence in 2013; stand count in 2014; and, stand count, core 1, and whitehead incidence in 2015. 

## Figures and Tables

**Table 1:** Statistical comparisons of the effects of variety (Var), Dermacor seed treatment (Derm), silicon amendment (Sil), and their interactions on rice water weevil larval densities. Core 1 and Core 2 sampling were performed three and four weeks after permanent flooding in 2013, 2014, and 2015.

Treatment	Larval Density											
	2013				2014				2015			
	Core 1		Core 2		Core 1		Core 2		Core 1		Core 2	
	*F*_1, 23_	*P*	*F*_1, 23_	*P*	*F*_1, 23_	*P*	*F*_1, 24_	*P*	*F_1_*_, 23_	*P*	*F*_1, 24_	*P*
Var	1.33	0.2607	0.14	0.7095	6.93	0.0149	5.47	0.0281	0.01	0.9117	0.61	0.4439
Derm	48.37	<0.0001	68.50	<0.0001	111.66	<0.0001	54.62	<0.0001	95.88	<0.0001	55.33	<0.0001
Sil	1.68	0.2077	2.25	0.1473	4.60	0.0427	1.46	0.2393	0.03	0.8591	0.00	1.000
Var × Der	10.65	0.0034	18.54	0.0003	0.72	0.4040	0.57	0.4581	8.57	0.0076	16.53	0.0004
Var × Sil	0.76	0.3908	0.39	0.5387	1.79	0.1946	0.00	1.000	0.01	0.9400	0.37	0.5506
Derm × Sil	0.39	0.5365	0.56	0.4631	6.93	0.0149	0.96	0.3367	2.90	0.1019	0.27	0.6085
Var × Derm × Sil	1.03	0.3214	0.08	0.7839	3.32	0.0815	0.46	0.5038	0.58	0.4523	0.19	0.6693

**Table 2:** Field activities and corresponding dates.

Activity	Year		
	2013	2014	2015
Seed Treatment	20 May	5 May	8 June
Planting	21 May	9 May	10 June
Silicon Slag Application	21 May	9 May	10 June
Stand Count	6 June	2 June	6 July
Permanent Flood	19 June	13 June	10 July
1st Core Sampling	10 July	8 July	31 July
2nd Core Sampling	17 July	15 July	7 August
Whitehead counts	16 August	12 August	14 September
Harvest	13 September	9 September	6 October
